# Identifying factors affecting age at first semen freezing and age at first semen use in Sahiwal bulls

**DOI:** 10.14202/vetworld.2015.928-931

**Published:** 2015-07-29

**Authors:** B. C. Naha, A. K. Chakravarty, M. A. Mir, V. Jamuna, A. P. Singh, D. Maher

**Affiliations:** 1 Division of Animal Genetics, Indian Council of Agricultural Research, Indian Veterinary Research Institute, Izatnagar, Bareilly, Uttar Pradesh, India; 2 Dairy Cattle Breeding Division, Indian Council of Agricultural Research, National Dairy Research Institute, Karnal, Haryana, India

**Keywords:** age at first semen freezing, age at first semen use, non-genetic factors, Sahiwal breeding bull

## Abstract

**Aim:**

The objective of the study was to evaluate the effects of non-genetic factors on reproduction traits *viz*. age at first semen freezing and age at first semen use of breeding bulls in Sahiwal bulls by fitting least-squares analysis.

**Materials and Methods:**

The information on reproduction traits of 43 Sahiwal breeding bulls belonging to 8 sets of Sahiwal breeding program at Indian Council of Agricultural Research-National Dairy Research Institute (ICAR-NDRI), Karnal (Haryana), India during 27 years (1987-2013) were analyzed using fixed linear model. The information was collected from AI records, reproduction sheets, and bull AI register maintained at different sections of Institute *viz*. record room of Dairy Cattle Breeding Division (DCB), Cattle Yard, Artificial Breeding Research Centre, ICAR-NDRI, Karnal.

**Results:**

The average age at first semen freezing and age at first semen use of Sahiwal breeding bulls was estimated as 3.17±0.01 years and 5.35±0.01 years, with the coefficient of variation 18.93% and 20%, respectively. The overall least-squares mean for age at first semen freezing and age at first semen use was estimated as 3.14±0.09 years and 5.25±0.02 years, respectively, in Sahiwal breeding bulls. Period of freezing/use had significant effects on reproductive traits (p<0.01). Season had no significant effect on any of the traits considered in this study.

**Conclusion:**

It can be concluded that management inputs such as nutrition, breeding, and optimum environment should be taken care of to optimize age at first semen freezing and age at first semen use for better utilization of superior germplasm.

## Introduction

Livestock is a very important subsector of Indian agricultural production system. The overall contribution of the livestock sector in total GDP is almost 4.11% [1]. India ranks first in milk production in the world (132.4 million tonnes), which is mostly contributed by cattle and buffalo [2]. Among the milk producing cattle, Sahiwal is distinctly the best indigenous dairy cattle breed of India having high merit in economic traits.

The bull has a high economic value attached with it and thus need to be maintained on proper nutrition and management to obtain optimum performance in terms of semen production. The demand for the best males has increased considerably due to a shortage in the number of proven bulls having better semen characteristics for sustaining a successful breeding program [3]. The reproductive parameters being quantitative in nature are affected by genetic as well as non-genetic factors [4]. Actual fertility of a herd is a result of the genetic potential and environmental factors including nutrition, health, and management of cows and bulls [5]. However, when we talk of the environment, nutrition and management goes with it and only one aspect cannot be taken in isolation.

Bull’s feeding generally is not given due importance, as a result, its age at puberty and first semen collection get delayed. The differences in bull management studies have shown variation in fertility of the herd [6]. Age also affects semen production as reported by Mandal *et al*. [7]. The availability of semen at the earliest possible age from breeding bulls is not only economical but also may increase productive life span and proving the bulls under progeny testing program [8]. A bull’s highest fertility has been observed at around 2-4 years of age and started declining once bull attained more than 4 years of age [9].

There is little information on the effects of non-genetic factors on reproduction traits of Sahiwal breeding bulls particularly age at first semen freezing and age at first semen use. Hence, the present study was carried to look into these aspects of Sahiwal bull production.

## Materials and Methods

### Ethical approval

The present study was approved by Institutional Animal Ethics Committee of National Dairy Research Institute.

### Overview

Age at first semen freezing and age at first semen use represent the age when the semen is collected for first freezing and the time it is first used in the herd. Under the Sahiwal progeny testing program, bulls are mainly judged based on the daughter’s performance in the herd. Minimum 8-10 bulls in each set are used in Sahiwal breeding program, and the set duration of each set (test cycle) is around 24 months. In the present study, 8 sets of Sahiwal bulls were evaluated with a minimum number of three bulls in III and IV set and maximum number of eight bulls in X set at NDRI herd.

### Location of study

The study was carried out at Artificial Breeding Research Centre (ABRC), ICAR-National Dairy Research Institute (NDRI), Karnal, Haryana, India. The farm is 250 m above the mean sea level on latitude 29.43 N and longitude 77.2 E. The climate is subtropical in nature. The atmospheric temperature varies from about 0°C in winter months to about 45°C in summer months. The average annual rainfall is approximately 760-960 mm, mostly during the months of July and August. Relative humidity varies from 41% to as high as 85%.

### Sample population and classification of data

The present study was conducted on 43 Sahiwal breeding bulls belonging to 8 sets of Sahiwal progeny testing project maintained at Artificial Breeding Research Centre ICAR-NDRI, Karnal. The traits under study were age at first semen freezing and age at first semen use. The data were classified into various sub-classes as, season/period of freezing for age at first semen freezing and season/period of use, parity, stages of lactation, and age of Sahiwal cows for age at first semen use. To evaluate the effect of various non-genetic factors on reproduction traits of breeding bulls, the data were grouped into different classes based on period, season, parity, stage of lactation, and age of Sahiwal cows. The data were classified into 8 periods *viz*. Period I (1987-1992); Period II (1993-1996); Period III (1996-1999); Period IV (1999-2002); Period V (2002-2004); Period VI (2004-2007); Period VII (2007-2010); and Period VIII (2010-2013). Based on season of AI, each year was classified into four major seasons *viz*., winter (December to March), summer (April to June), rainy (July to September), and autumn (October to November), depending on prevalent meteorological factors as recorded in CSSRI, Karnal [10]. Based on different lactation, parity was classified as I, II, III, IV, and V lactation and above. The stage of lactation was classified as Stage I (0-90 days); Stage II (90-120 days); and Stage III (120 days and above).

### Statistical analysis

Mean and standard error of the reproductive traits were calculated using standard statistical procedure [11]. On standardization and normalization of traits, the number of bulls remained in the ­analysis were 41 for age at first semen freezing and 40 for age at first semen use. The least-squares procedures, as described by Harvey [12], were used to analyze the data. Furthermore, Duncan’s multiple range test, as modified by Kramer [13], was used for testing the differences among least-squares means (using inverse coefficient matrix) between subclasses of periods, seasons, parity, and stage of lactation. The following models were used with assumptions that different components being fitted into the model were independent and additive.

The model used for age at first semen freezing of Sahiwal bulls was as follows:


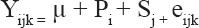


where, Y_ijk_ is the observation on the k^th^ bull in i^th^ period, j^th^ season; m is the overall mean; P_i_ is the effect of i^th^ period (1-8); S_j_ is the effect of j^th^ season (1-4); e_ijk_ is the random error ~ NID (0, σ^2^_e_).

The model considered for age at first semen use of Sahiwal bulls as follows:


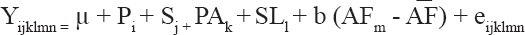


where, Y_ijklmn_ is the observation on the n^th^ bull in i^th^ period, j^th^ season, k^th^ parity, l^th^ stages of lactation, and m^th^ age of cow; m is the overall mean; P_i_ is the effect of i^th^ period (1-8); S_j_ is the effect of j^th^ season (1-4); PA_k_ is the effect of k^th^ parity (1-5 and above); SL_l_ is the effect of l^th^ stage of lactation (1-3); b is the regression of age of female on the Age at first semen use; AF_m_ is the age of m^th^ cow; 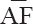 is the average age of the cow e_ijklmn_ is the random error ~ NID (0, σ^2^_e_).

## Results and Discussion

The present study was not to observe the influence of non-genetic factors on seminal parameters.

**Figure-1:**
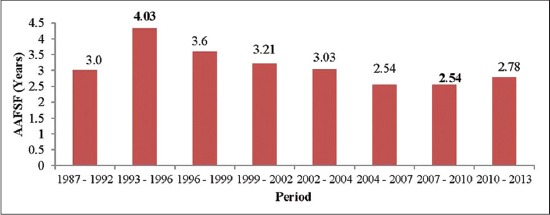
Period wise variation of age at first semen freezing of Sahiwal bulls, AAFSF=Age at first semen freezing.

Freezing of semen is an important criterion for evaluating a bull, and the aim is to get sufficient number of frozen semen doses from a Sahiwal bull at the beginning of the set [14]. The main target of using frozen semen for AI in breeding program is to use the bulls randomly i.e. all bulls must have almost the same number of AI at completion of set and all bulls must be used from the beginning of the set [14]. The coefficient of variation for average age at first semen freezing of Sahiwal bulls varied in different sets with a minimum of 5.5% in Period VI to maximum of 23.17% in Period I. The overall least-squares mean of age at first semen freezing was estimated as 3.14±0.09 years in Sahiwal bulls. The mean obtained was similar with the values reported by Mukhopadhyay *et al*. [4] and lower as compared to values reported by Khatun *et al*. [15] in Sahiwal bulls. Lower mean of 1101±29 days was reported by Chauhan *et al*. [16] in Karan Fries bulls and 865.72±34.60 days by Thippeswamya *et al*. [17] in crossbred bulls. The analysis of variance for season/period of freezing, season/period of use, stage of lactation, age of cows, and parity affecting age at first semen freezing and age at first semen use are presented in Table 1. Only period of freezing had a significant effect (p<0.01) on age at first semen freezing (Table-1 and Figure-1). Similar findings were reported by other workers in Sahiwal bulls [4]. Season of freezing had no significant influence on the age at first semen freezing, which is in consonance with the finding reported in Karan Fries bulls by Chauhan *et al*. [16] and in Ongole bulls by Bhakat *et al*. [18].

**Table-1:** Analysis of variance (MS values) of age at first semen freezing and age at first semen use of Sahiwal bulls.

Sources of variation	Mean sum of squares	

	AAFSF (years)	AAFSU (years)
Period of freezing/use	1.20** (7)	203.27** (6)
Season of freezing/use	0.19 (3)	0.31 (3)
Parity	-	0.26 (4)
Stages of Lactation	-	1.77 (2)
Age of female	-	0.14 (1)
Error	0.25 (30)	0.64 (23)

Figures in parentheses indicate respective degrees of freedom.

** p<0.01, AAFSF=Age at first semen freezing,

AAFSU=Age at first semen use

**Figure-2:**
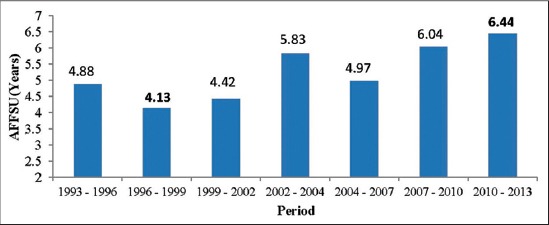
Period wise variation of age at first semen use of Sahiwal bulls, AAFSU=Age at first semen use.

The coefficient of variation for the average age at first semen use of Sahiwal bulls varied in different sets with a minimum of 8.14% in Period V to a maximum of 23.24% in Period VI. The overall least-squares mean for age at first semen use was estimated as 5.25±0.02 years for Sahiwal bulls. No literature is available regarding the age at first semen use of Sahiwal bulls under progeny testing program to compare our data with others’ finding. The overall least-squares means for age at first use was estimated as 3.96±0.03 years in Murrah bulls [14]. Period of use had a significant effect (p<0.01) on age at first use of Sahiwal bulls (Table-1 and Figure-2). The effect of parity, stage of lactation, and age of Sahiwal cows were found non-significant in age at first semen use. Till now, no literature is available regarding the effect of these non-genetic factors on age at first semen use in Sahiwal bulls. Period (p<0.01) and season (p<0.05) of the use of bulls had a significant effect on age at first use of Murrah breeding bulls [14].

In the first five periods i.e. April 1987 - July 2004, the age at first semen freezing was higher; however, after that it declined (Figure-1) which indicates better nutritional management of Sahiwal bulls as silage based feeding system was replaced by a more efficient energy based feeding system from December 2003 in the NDRI herd. In contrast to above findings, the age at first semen use was lower during the first three periods, i.e. April 1993 - March 2002, whereas after that it was found to be increasing (Figure-2). The inconsistency in the age at first semen use in different periods may be due to set duration of Sahiwal bulls as set duration varies from one set to another set under progeny testing program. Use of the bulls in a particular set may sometimes take longer duration depending upon the production of frozen semen doses by the bulls and to cover the same number of AI for all bulls at the completion of the set. Therefore, age at first semen use was not uniform. Reproductive traits of the breeding bulls are integral to achieve the stated objectives of the successful breeding program. The studies on the period generally involve temporal aspect (vertical classification of the data on period). In this study, the period was the sum total of changes in temporal and spatial (microenvironment) aspects and different periods basically represented the changes in management practices of breeding bulls.

## Conclusion

From an overview of the findings, it could be concluded that period of semen freezing/use had a favorable influence on the traits of breeding bulls. However, no consistent trend could be inferred for the influences of different non-genetic causes on the reproduction traits. The age at first semen freezing, and use of Sahiwal bull’s semen were significantly influenced by the period of semen freezing/use. Age at first semen freezing and age at first semen use of Sahiwal bulls were 3.14±0.09 and 5.35±0.01 years, respectively, which is emphasizing the need to take holistic management i.e. (better care, training, energy levels of nutrition, and other management practices) approach in more scientific lines to achieve early age at sexual maturity and age at first semen freezing to harvest more quality frozen semen for wider coverage of AI under progeny testing program. It can be emphasized that the holistic management can have an important bearing on reducing the cost of Sahiwal bull production in tropical and subtropical regions.

## Authors’ Contributions

AKC has planned the study. BCN recorded the information and analyzed the data. MAM, VJ, APS, DM provided help in the analysis of data. MAM drafted and revised the manuscript under the guidance of AKC. All authors read and approved the final manuscript.
